# An Unusual Case of Intraosseous Desmoplastic Fibroblastoma (Collagenous Fibroma) of the Mandible

**DOI:** 10.7759/cureus.75904

**Published:** 2024-12-17

**Authors:** Rana Al-Zaidi

**Affiliations:** 1 Laboratory and Blood Bank, Anatomic Pathology Section, King Faisal Hospital, Makkah, SAU

**Keywords:** bone neoplasms, collagenous fibroma, desmoplastic fibroblastoma, intraosseous, mandible

## Abstract

Desmoplastic fibroblastoma (collagenous fibroma) is a rare, benign fibroblastic/myofibroblastic tumor that usually arises in the subcutaneous and intramuscular tissues. This tumor type has rarely been described to arise primarily in bones. This is a case of intraosseous desmoplastic fibroblastoma occurring in the mandible of a 35-year-old woman that was detected incidentally during a routine dental examination. The patient underwent surgical excision of the lesion. Microscopic examination demonstrated a paucicellular fibro-collagenous spindle cell neoplasm that showed a fibroblastic/myofibroblastic immunophenotype, consistent with desmoplastic fibroblastoma of the bone. The case presents an extremely rare intraosseous manifestation of an uncommon soft tissue tumor, highlighting its diverse anatomical and compartmental distribution.

## Introduction

Desmoplastic fibroblastoma (also known as collagenous fibroma) is an uncommon fibroblastic/myofibroblastic soft tissue tumor first described by Evans in 1995 [1]. This tumor type is classified as a benign neoplasm according to the World Health Organization classification of tumors of soft tissue and bone, 2020 [2]. The typical clinical presentation is a slow-growing, painless mass of relatively long duration that affects adults of both genders, with a peak incidence in the fifth to seventh decades of life [3]. Desmoplastic fibroblastoma has a wide anatomical distribution, most commonly in the upper arm, shoulders, dorsal aspect of the body, and lower limbs. Uncommonly reported sites of involvement include the oral cavity, head and neck, axilla, breast, thyroid, cardiac valve, diaphragm, and anterior mediastinum [4-8]. Although most cases have been reported primarily in the subcutaneous tissue and skeletal muscle compartments, desmoplastic fibroblastoma has been observed in superficial dermal locations and deeper fascial tissues [5]. Primary bone tumors are rare, with only three cases reported in the medical literature [9-11]. The aim of this report is to present the fourth case of desmoplastic fibroblastoma, to the knowledge of the author, of the bone arising in the mandible, along with a medical literature review discussing the radiopathological characteristics and potential diagnostic pitfalls.

## Case presentation

**Figure 1:**
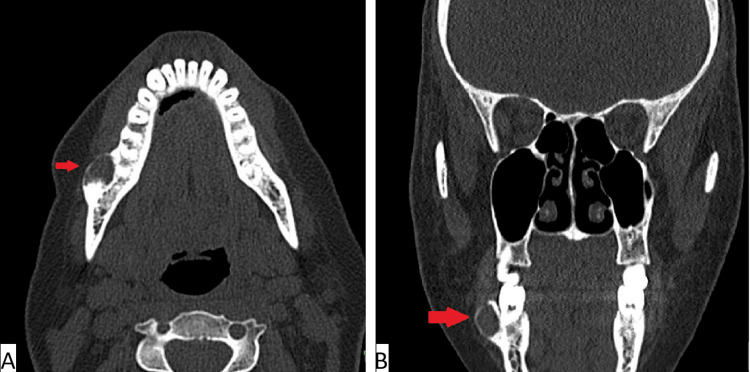
Noncontrast computed tomography photograph of maxillofacial bones demonstrating an oval, intra-osseous, unilocular, lytic lesion with sclerotic margins (red arrows), located posterolateral to the last right molar tooth. A: axial view;  B: coronal view.

A 35-year-old woman presented to a dental clinic for tooth extraction. During the routine dental evaluation, periapical radiolucency was discovered on an orthopantomogram (OPG) in relation to the right mandibular third molar tooth extending to the ramus. The patient did not report any pain or symptoms related to the lesion. She was a known case of subclinical hypothyroidism on medical therapy. On examination, the only abnormality seen was the expansion of the buccal cortical plate overlying the lesion. Regional lymph node enlargement was not observed. Non-contrast computed tomography (CT) scan revealed a 2x1.4x1.2 cm right mandibular intraosseous, oval, unilocular, lytic lesion with sclerotic margins, located posterolateral to the last molar tooth (Figure 1A, Figure 1B). Mild erosion of the superior margin was observed, along with remodeling of the adjacent bone. The radiological diagnosis was a periodontal or radicular cyst. Therefore, a surgical excision was performed.

**Figure 2:**
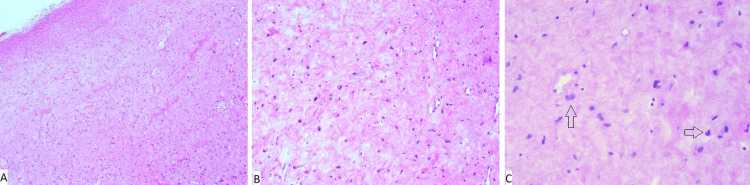
Microscopic appearance of desmoplastic fibroblastoma of the bone. A: Low-power view depicting a well-circumscribed, hypocellular, collagenous lesion with inconspicuous vasculature (hematoxylin-eosin, original magnification x40). B: On x100 magnification, the lesion is composed of bland stellate to spindled fibroblasts, set in a collagenous background. Few reactive-appearing cells with smudgy nuclei are seen (hematoxylin-eosin, original magnification x100). C: Several bi-nucleated cells are depicted (arrows) (hematoxylin-eosin, original magnification x200).

Grossly, the resected specimen consisted of three pieces of homogeneously white-pearly firm tissue measuring 2x1.5x1 cm in aggregate. Microscopic examination of the totally submitted specimen revealed a well-demarcated, hypocellular lesion comprising haphazardly arranged, reactive-appearing, spindled to ovoid cells (Figure 2A). They showed elongated to oval-shaped bland-appearing nuclei with fine to focally smudgy chromatin patterns, inconspicuous small nucleoli, smooth nuclear contours, and scant amphophilic cytoplasm. A subset of lesional cells showed bi-nucleation (Figure 2B, Figure 2C).

**Figure 3:**
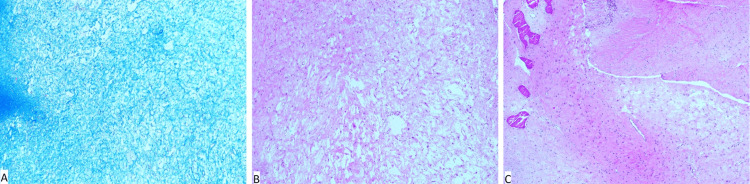
Microscopic appearance of desmoplastic fibroblastoma of the bone. A: The collagenous stroma is highlighted with Masson trichrome staining (original magnification x20). B and C: Focal edematous zones and entrapment of reactive woven bone at the periphery are depicted (hematoxylin-eosin, original magnifications x40).

The spindle cells were embedded in a collagenous stromal background of variable density (highlighted with trichrome staining) and demonstrated multifocal edematous zones (Figure 3A, Figure 3B). Sparse, small-caliber, thin-walled blood vessels and sprinkles of mast cells and small lymphocytes were observed. Focal entrapment of the reactive woven bone trabeculae was noted at the tumor edge (Figure 3C). The lesion lacked mitotic figures, hemorrhage, and necrosis. Importantly, no intralesional bone trabeculae or osseous components were observed. 

**Figure 4:**
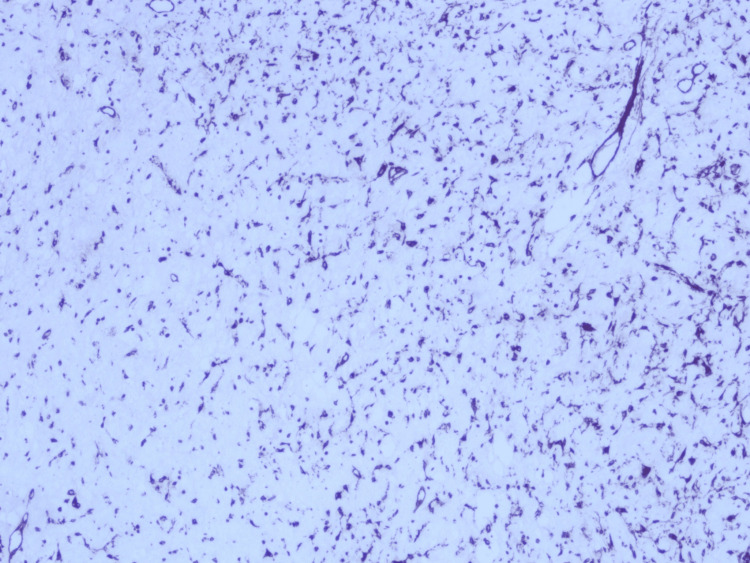
Strong and diffuse reactivity with vimentin was seen. Immunohistochemistry, original magnifications x40.

Immunohistochemical studies demonstrated diffuse and strong expression of vimentin, whereas staining for smooth muscle actin (SMA) showed only weak and focal reactivity (Figure 4). The spindle cells did not express desmin, CD34, and S100 protein and showed a low Ki-67 proliferation index. Based on these findings, the diagnosis was that of a desmoplastic fibroblastoma (collagenous fibroma) of bone/primary intraosseous desmoplastic fibroblastoma. The patient was doing well postoperatively, with no evidence of tumor recurrence after nine months of regular follow-up.

## Discussion

Desmoplastic fibroblastoma (collagenous fibroma) is a rare, benign, fibrous soft tissue tumor with fewer than 150 reported cases since the first report in 1995. Desmoplastic fibroblastoma affects a wide age range, mostly adults, with a peak incidence in the fifth to seventh decades of life and slight male gender predominance [3]. Clinical manifestations generally depend on the tumor size and anatomic location. Desmoplastic fibroblastoma can be asymptomatic or present with nonspecific symptoms, often as a painless, slow-growing mass of generally long duration that is not usually associated with a history of trauma. Presentation with pain or neurological symptoms related to its location is a rare feature [3,12]. This particular tumor is characterized by a wide anatomical distribution in the head and neck, oral cavity, upper and lower extremities, and trunk [1-5]. Two-thirds of cases are located subcutaneously, and one-third involve the skeletal muscles or deep soft tissues [3].

Desmoplastic fibroblastoma of soft tissue often appears on gross examination as a well-circumscribed rounded-ovoid firm mass with a smooth white outer surface and a homogenous white pearly-gray-tan cut surface. Cystic-like areas may be observed, particularly in larger tumors [1,3]. Microscopic examination typically shows hypocellular proliferation of bland spindled-stellate fibroblasts/myofibroblasts with oval-rounded nuclei, small eosinophilic nucleoli, and a variable amount of amphophilic cytoplasm. Infrequently, bi-nucleated cells and floret-like multinucleated giant cells are observed [3,13,14]. Spindle cells are sparely distributed in a generally dense fibrillar collagenous to myxo-collagenous stroma featuring scant vasculature and few if any, inflammatory cells. Degenerative changes such as dystrophic calcification and metaplastic bone formation are observed occasionally [13]. Desmoplastic fibroblastoma lacks significant nuclear atypia, mitotic activity, fascicular growth patterns, stromal hemorrhage, and necrosis. Although desmoplastic fibroblastoma of soft tissue often appears well-circumscribed and unencapsulated on low-power magnification, occasional cases feature ill-defined borders with peripheral focal entrapments of adjacent structures, such as adipose tissue, collagen fibers, skeletal muscle, and nerve fibers [1,3,5]. Indeed, involvement or infiltration of adjacent bone with destruction has also been described, although infrequently, highlighting the potential multi-tissue involvement of this unique tumor [15-17].

In contrast, primary intraosseous localization is an extremely rare phenomenon. A literature review in the PubMed, MEDLINE, and ScienceDirect databases for all relevant English-language full texts and abstracts using the search terms "desmoplastic fibroblastoma/collagenous fibroma" and "bone/intraosseous" identified only three cases of primary desmoplastic fibroblastoma of bone (Table 1). Three of the cases, including this one, were found in female patients; in contrast, male predominance was observed in soft tissue tumors [3,9-11]. Two of the primary desmoplastic fibroblastomas of the bone, as in this case, arose in the oral cavity, while the other was in the proximal femur. The clinical presentation of these cases was an incidental finding during routine medical evaluations [9-11]. Radiologically, primary bone tumors are characterized by a well-demarcated, radiolucent appearance with a variably thickened sclerotic rim and are devoid of radiodense areas that would suggest matrix production or hemorrhage. They show low signal intensity on T1-weighted MRI images and slightly high signal intensity on T2-weighted images, suggesting a collagen-rich tumor. Additionally, an absence of enhancement was noted in images following contrast media injection [9-11]. Primary desmoplastic fibroblastoma of the femur was treated with curettage, whereas the two cases of maxillary bone were excised surgically, as in the current case. The resected tumors measured 1.5-2 cm in the greatest dimension and were either fragmented or intact rounded masses with smooth white-gray outer surfaces and solid cut sections with some cystic-like areas [9-11]. Microscopically, morphological features seen in this case are almost identical to those described in the previously reported cases of primary desmoplastic fibroblastoma of the bone. Indeed, the desmoplastic fibroblastoma of bone shows microscopic features analogous to those of soft tissue desmoplastic fibroblastoma. Immunohistochemical analysis of primary desmoplastic fibroblastoma of the bone showed expression similar to that of soft tissue tumors, including consistent diffuse positivity for vimentin and focal to absent reaction for smooth muscle markers, such as SMA [3,9-11].

**Table 1:** Clinical characteristics of desmoplastic fibroblastoma/collagenous fibroma reported primarily in bone.

Case number	References	Age (years), gender	Clinical presentation	Radiological features of the lesion	Anatomic location	Size (cm)	Treatment	Follow-up, clinical outcome
1	Gong et al., 2018 [9]	46, male	Not available	Osteolytic with thick sclerotic rim	Proximal femur	1.5	Curettage	1 year, no recurrence
2	Jaafari-Ashkavandi et al., 2018 [10]	58, female	Incidental finding	Well-defined radiolucency	Maxillary alveolar bone	2	Surgical excision	1 year, no recurrence
3	Garcia et al., 2018 [11]	43, female	Incidental finding	Well-defined radiolucency	Maxillary edentulous area, left side	2	Surgical excision	6 months, no recurrence
4	Present case	35, female	Incidental finding	Osteolytic with sclerotic rim	Mandible, right side	2	Surgical excision	9 months, no recurrence

The most important differential diagnostic consideration for primary intraosseous desmoplastic fibroblastoma is desmoplastic fibroma of the bone, a rare, locally aggressive bone tumor that most commonly affects the posterior aspect of the mandible and long tubular bones [18,19]. Desmoplastic fibromas usually present with pain or a palpable mass, and in some cases, tumors are asymptomatic and discovered incidentally, similar to intraosseous desmoplastic fibroblastoma. Radiologically, desmoplastic fibromas of the bone appear as a radiolucent, lobulated, and expansile tumor with well-defined to ill-defined margins, often with internal trabeculation, a non-sclerotic rim, and cortical bone expansion or destruction. On MRI, these tumors appear as osteolytic lesions with hypointense to isointense signals on both T1 and T2 weighted images. Heterogeneous signal enhancement is a characteristic feature of desmoplastic fibromas of the bone [18,19]. This finding, in particular, would be valuable as a distinguishing feature of the desmoplastic fibroma of the bone, as intraosseous desmoplastic fibroblastoma does not show any signal enhancement following intravenous contrast media administration [9]. Unlike intraosseous desmoplastic fibroblastoma, microscopic examination of desmoplastic fibroma typically shows interlacing, hypocellular to moderately cellular fascicles of uniform bland spindle cells embedded in an abundant fibrillar collagenous stroma with scant vascularity, often intermingled with thin spicules of reactive bone trabeculae [18,19].

Desmoplastic fibromas of the bone typically show diffuse and strong immunohistochemical expression of vimentin and focal positivity for SMA, similar to desmoplastic fibroblastoma. However, they lack specific immunohistochemical markers to reliably distinguish them from their mimics [19]. Strong and diffuse overexpression of the nuclear transcription factor* FOSL1* has been recently identified in 80% to 100% of soft tissue desmoplastic fibroblastoma, which is regarded as a specific feature of this tumor type. This is supported by the identification of somatic rearrangements in *FOSL1* in most tumors. This would be of great utility in rare and challenging cases, although conventional microscopy alone is considered sufficient to reach a diagnosis of desmoplastic fibroblastoma in most cases [20,21]. Accurate diagnosis and recognition of desmoplastic fibroblastoma of the bone are of great importance because the treatment of choice for this tumor is simple surgical excision, whereas wider resection is recommended for desmoplastic fibroma owing to the locally aggressive nature of this tumor type [1,3,19]. It would be appropriate to give the name "collagenous fibroma of bone/intraosseous collagenous fibroma" to primary bone tumors rather than "desmoplastic fibroblastoma of bone" to avoid potential confusion with the aggressive tumor "desmoplastic fibroma of bone."

Desmoplastic fibroblastoma can be misdiagnosed radiologically with other mandibular lesions such as odontogenic keratocyst and ameloblastoma. Odontogenic keratocyst, a benign developmental odontogenic cyst, appears as a well-circumscribed unilocular or multilocular radiolucency with uniform sclerotic borders and occasional cortical expansion, often associated with an unerupted tooth. Histopathologically, odontogenic keratocyst is characterized by a cystic space lined by a uniform para keratinized stratified squamous epithelium of 5-10 cell layers, with a distinct basal layer of palisaded cuboidal to columnar cells [22]. Ameloblastoma, a benign, locally aggressive odontogenic epithelial tumor, presents radiologically as a well-defined, expansile, unilocular or multilocular radiolucency with a characteristic honeycomb or soap-bubble appearance, surrounded by a radiopaque margin and often causing expansion of the bone cortex. Histopathologically, ameloblastoma resembles the enamel organ of a developing tooth with islands and cords of epithelium in a fibrous stroma [23]. Desmoplastic fibroblastoma generally follows a benign clinical course with no local recurrence or distant metastasis. Nevertheless, an extremely rare incidence of local recurrence was documented in one case of soft tissue desmoplastic fibroblastoma [20].

## Conclusions

Desmoplastic fibroblastomas are uncommon benign fibroblastic/myofibroblastic soft tissue tumors that can rarely arise primarily in bones. Accurate diagnosis can be achieved by the correlation between clinical findings and characteristic histopathological features, along with the judicial use of immunohistochemical staining. The precise histological diagnosis is necessary to guide clinical management and avoid aggressive treatment.
